# Exercise Modulates the Osteoimmune Axis: Myokine‐Mediated Mechanisms in Diabetic Bone Disease

**DOI:** 10.1002/dmrr.70245

**Published:** 2026-10-26

**Authors:** Chao Wu, Jun Zou, Yu Yuan

**Affiliations:** ^1^ School of Sports and Health Shanghai University of Sport Yangpu Shanghai China; ^2^ School of Exercise and Health Guangzhou Sport University Guangzhou Guangdong China

**Keywords:** bone remodelling, exercise, myokines, osteoimmunology, T2DM

## Abstract

Patients with type 2 diabetes mellitus (T2DM) often present with normal or even elevated bone mineral density (BMD), yet exhibit a significantly increased fracture risk—a phenomenon referred to as the ‘diabetic bone paradox’. This discrepancy underscores the inadequacy of conventional BMD‐centred assessment systems in fully elucidating the mechanisms underlying bone fragility in T2DM. Current evidence suggests that T2DM‐related skeletal fragility is multifactorial, involving alterations in bone quality, advanced glycation end‐product (AGE) accumulation, low bone turnover, microarchitectural deterioration, diabetic complications, increased fall risk and chronic inflammation. Recent advances in osteoimmunology have revealed that immune cell polarization driven by chronic low‐grade inflammation, along with an imbalanced receptor activator of nuclear factor‐κB ligand/osteoprotegerin (RANKL/OPG) axis, may contribute to the dysregulation of bone remodelling in T2DM. As a cornerstone intervention in T2DM management, exercise not only acts directly on bone tissue via mechanical loading but also may exert systemic immunomodulatory effects through myokines secreted by skeletal muscle. Exercise‐induced myokines, such as interleukin‐6 (IL‐6), irisin, β‐aminoisobutyric acid (BAIBA) and interleukin‐15 (IL‐15), may modulate the osteoimmune microenvironment and restore the dynamic equilibrium between bone resorption and formation by modulating macrophage polarization, restoring the Th17/Treg balance, bone marrow adipose tissue metabolism, and chronic inflammatory signalling pathways. Moreover, exercise may further promote bone metabolic homoeostasis by suppressing negative regulatory signals, such as myostatin. Therefore, this narrative review summarises the aberrant features of the osteoimmune axis in T2DM, with a focus on the potential mechanisms by which exercise and its induced myokines may modulate the osteoimmune microenvironment to ameliorate diabetic bone disease, thereby providing a framework for understanding exercise as a potential non‐pharmacological intervention and identifying areas requiring further translational investigation.

## Graphical abstract

Exercise activates skeletal muscle to release myokines (irisin, acute IL‐6, IL‐15 and BAIBA) and suppress myostatin, which may help counteract T2DM‐induced hyperglycemia, AGE accumulation, chronic inflammation and osteoimmune imbalance, potentially contributing to improved bone quality and reduced fracture risk.

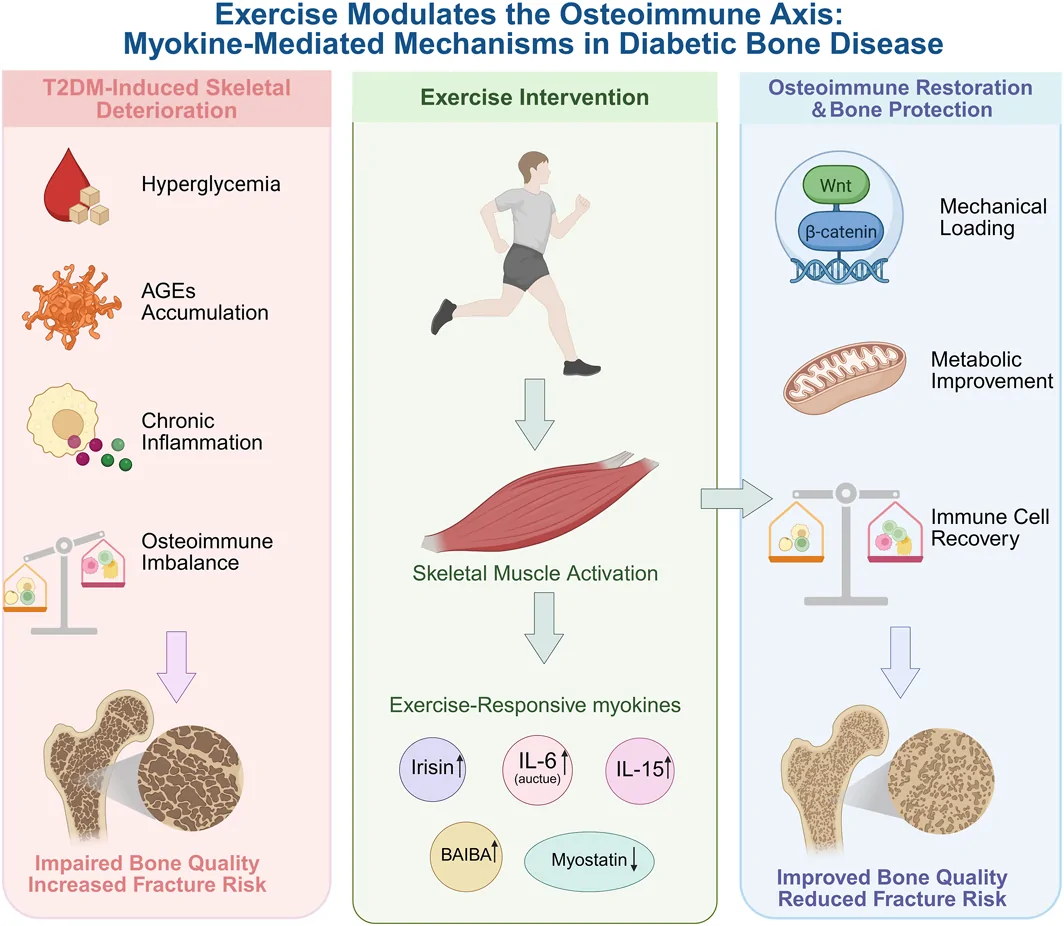

## 1 Introduction

Type 2 diabetes mellitus (T2DM), the most prevalent form of diabetes, is a metabolic disease characterised by chronic hyperglycemia and has become one of the fastest‐growing chronic diseases worldwide. Epidemiological data indicate that there were 529 million cases of DM globally in 2021, and this number is projected to rise to 1.3 billion by 2050 [1]. Beyond cardiovascular, neurological, and renal complications, skeletal involvement has gained increasing attention in recent years [2]. A recent meta‐analysis of over 13 million individuals reported that T2DM was associated with a 25% higher overall fracture risk, with stronger associations in women and individuals with longer diabetes duration [3]. Similarly, another meta‐analysis demonstrated elevated risks of osteoporosis and fractures in T2DM, though with substantial heterogeneity across populations and fracture sites [4]. Diabetic bone disease refers to a spectrum of skeletal abnormalities associated with T2DM, including impaired bone quality, altered bone remodelling, increased skeletal fragility, and elevated fracture risk [5]. In particular, patients with T2DM often exhibit normal or even elevated bone mineral density (BMD) but paradoxically have a significantly increased risk of hip and vertebral fractures—a phenomenon termed the diabetic bone paradox [6]. Moreover, post‐fracture mortality risk is substantially elevated in patients with T2DM [7]. Current evidence suggests that T2DM‐related skeletal fragility is multifactorial, involving alterations in bone quality, advanced glycation end‐products (AGEs) accumulation, low bone turnover, microarchitectural deterioration, diabetic complications, increased fall risk, and chronic inflammation [8, 9]. While these factors collectively contribute to diabetic bone disease, growing attention has recently been directed towards the role of osteoimmune dysregulation as a potential mechanistic link between metabolic dysfunction and impaired skeletal homoeostasis.

Osteoimmunology, an interdisciplinary field that examines the shared molecular and cellular pathways between the skeletal and immune systems, provides a valuable framework for investigating this connection [10]. The skeletal and immune systems share multiple cell populations and signalling pathways during development and homoeostasis, with receptor activator of nuclear factor‐κB ligand (RANKL) serving as a pivotal molecule linking these two systems [11]. In the context of T2DM, evidence from animal models and in vitro studies has suggested that metabolic disturbances are associated with alterations in immune‐cell function and osteoimmune signalling pathways [12, 13, 14], including dysregulation of the RANKL/osteoprotegerin (OPG) axis, which may contribute to altered bone remodelling. However, direct evidence from human T2DM bone tissue, apart from a few small studies in alveolar bone [15], remains scarce, and most proposed mechanisms are supported primarily by preclinical findings, highlighting the need for further translational research. Although current anti‐osteoporotic therapies, such as bisphosphonates and denosumab, are clinically available, their suboptimal utilization in patients with T2DM and inability to directly target the underlying inflammatory and metabolic disturbances limit their capacity to fully address skeletal fragility in this population [16]. Consequently, interventions targeting the osteoimmune axis have emerged as an area of growing interest.

Exercise is widely recognized as a cornerstone intervention in the management of T2DM. Evidence from clinical trials and observational studies suggests that exercise may improve skeletal health at multiple levels, including modulation of bone turnover markers [17], preservation of bone mass [18], and reduction of fracture risk [19, 20]. Beyond the direct stimulation of bone tissue by mechanical loading, exercise also exerts systemic anti‐inflammatory effects [21]. Skeletal muscle is increasingly recognized as an important endocrine organ and secretes a variety of myokines, such as irisin, interleukin‐6 (IL‐6), and interleukin‐15 (IL‐15), during contraction [22]. These myokines act as important messenger molecules that may directly or indirectly modulate immune cell function and thereby influence the diabetic osteoimmune microenvironment [23]. The skeletal and myokine responses to exercise depend on modality, intensity, and duration, with aerobic, resistance, and combined training eliciting distinct effects [24]. However, the underlying molecular mechanisms remain incompletely understood.

Therefore, this narrative review focuses on T2DM as a specific pathological context and, from an osteoimmunological perspective, discusses current evidence regarding osteoimmune dysregulation under diabetic conditions. It further highlights how exercise and exercise‐induced myokines may reshape the osteoimmune microenvironment and promote homoeostatic osteoimmune balance to ameliorate diabetes‐related bone fragility. Unlike previous reviews that primarily summarise the direct skeletal effects of individual myokines, this review emphasises the exercise–myokine–osteoimmune axis as an integrated mechanistic framework, with particular attention to immune regulation within the bone marrow microenvironment. We hope this perspective will provide a theoretical basis for non‐pharmacological interventions in T2DM‐related bone disease and expand the translational potential of exercise as an immunomodulatory strategy in metabolic bone diseases.

## 2 T2DM‐Induced Osteoimmune Dysregulation in the Bone Microenvironment

### 2.1 Osteoimmune Dysregulation in the Bone Microenvironment Induced by T2DM‐Related Metabolic Abnormalities

T2DM‐associated skeletal fragility develops within a complex metabolic and inflammatory environment. Unlike primary osteoporosis, diabetic bone disease is characterised not only by alterations in bone mass but also by impaired bone quality, disturbed remodelling dynamics, and disrupted communication among metabolic, immune, and skeletal systems. Multiple pathological factors, including chronic hyperglycemia, insulin resistance, dyslipidemia, obesity, AGE accumulation, microvascular dysfunction, and altered muscle–bone signalling, may contribute to osteoimmune imbalance. In this section, the review focuses on hyperglycemia, AGE accumulation, and skeletal muscle secretory dysfunction as representative metabolic disturbances involved in T2DM‐related osteoimmune dysregulation.

#### 2.1.1 Hyperglycemia and Oxidative Stress

Chronic hyperglycemia represents a major metabolic stressor contributing to skeletal deterioration in T2DM [25]. Evidence from diabetic animal models and in vitro studies suggests that high glucose conditions impair bone remodelling through multiple mechanisms. Hyperglycemia may divert osteogenic progenitor cells towards adipogenic differentiation, promote a pro‐inflammatory bone marrow microenvironment, and impair trabecular mineralisation [26]. In parallel, hyperglycaemic and lipotoxic conditions suppress SIRT1 signalling and activate the p66Shc/ROS/NF‐κB pathway in osteoclast precursors, thereby enhancing RANKL signalling and increasing the RANKL/OPG ratio in vitro [27]. Hyperglycemia‐induced cellular stress may also promote osteocyte senescence and senescence‐associated secretory phenotype (SASP) formation, while impairing osteoblast function through endoplasmic reticulum stress and mitochondrial dysfunction [28, 29].

Excessive glucose metabolism through the polyol pathway, protein kinase C pathway, and mitochondrial electron transport chain increases reactive oxygen species (ROS) production, potentially exceeding nuclear factor erythroid 2‐related factor 2 (Nrf2)‐regulated antioxidant capacity [30, 31]. Elevated ROS activate FOXO1/FOXO3a, redistributing β‐catenin away from TCF/LEF‐mediated transcription towards FOXO‐dependent pathways and attenuating Wnt/β‐catenin signalling [32]. Given the essential role of Wnt signalling in osteogenesis, this mechanism may contribute to impaired bone formation under diabetic conditions. Moreover, oxidative stress promotes TNF‐α and IL‐6 production through NF‐κB activation, thereby facilitating osteoclastogenesis and bone resorption [33].

Thus, hyperglycemia‐induced oxidative stress may link metabolic dysfunction with osteoimmune dysregulation by impairing bone cell function and enhancing inflammatory signalling within the bone marrow microenvironment.

#### 2.1.2 Accumulation of AGEs

AGEs are heterogeneous molecules generated through non‐enzymatic glycation of proteins, lipids, and nucleic acids, with their accumulation enhanced under chronic hyperglycemia and oxidative stress [34]. In T2DM, excessive AGE accumulation contributes to skeletal fragility through structural deterioration and abnormal cellular signalling [35, 36].

Experimental studies in db/db and streptozotocin‐induced diabetic mouse models demonstrate that AGEs disrupt collagen cross‐linking, impair intrafibrillar mineralisation, and reduce bone strength [37]. Human studies further support these findings: femoral neck trabecular bone specimens from individuals with T2DM show increased pentosidine levels associated with reduced bone toughness despite preserved bone volume and mineralisation [38]. Femoral head specimens from postmenopausal women with T2DM undergoing hip arthroplasty demonstrate increased AGE content measured by HPLC, accompanied by elevated sclerostin (SOST), reduced RUNX2 expression, and impaired microarchitecture [39]. Similarly, iliac crest biopsies reveal increased pentosidine and fluorescent AGE accumulation in trabecular and cortical compartments as assessed by HPLC and multiphoton microscopy [40].

AGE accumulation is also associated with impaired osteogenic signalling, including reduced WNT10 B and LEF1 expression and decreased bone strength [41]. Recent evidence from a human study further demonstrates increased TNF‐α, reduced IL‐10 expression, suppressed Wnt signalling, and impaired bone strength in T2DM [42]. Beyond matrix effects, AGE–receptor for AGEs (RAGE) signalling contributes to osteoimmune dysregulation. RAGE is expressed in monocytes/macrophages, lymphocytes, and osteoclast precursor cells [43, 44]. AGE–RAGE activation induces NF‐κB signalling, promotes macrophage M1 polarization through Notch/NICD/RBP‐J signalling, and enhances osteoclastogenic activity [45, 46]. The resulting increase in pro‐inflammatory cytokines, including TNF‐α and IL‐6, may further stimulate RANKL‐dependent osteoclast differentiation and bone resorption, thereby promoting a pro‐resorptive bone marrow microenvironment.

Thus, excessive AGE accumulation may promote diabetic skeletal fragility through interconnected effects on bone material properties, Wnt‐dependent osteogenesis, and chronic inflammatory responses within the bone marrow microenvironment.

#### 2.1.3 Skeletal Muscle Secretory Dysfunction

Insulin resistance is a central feature of T2DM, and skeletal muscle represents a major metabolic target tissue [47]. Impaired insulin signalling reduces glucose transporter type 4 (GLUT4) translocation and glucose utilization, increases protein catabolism, and contributes to muscle loss and functional decline [48, 49]. Chronic metabolic stress further promotes myofiber atrophy, lipid accumulation, and impaired contractile function [50, 51].

Beyond these abnormalities, T2DM may induce pathological remodelling of skeletal muscle endocrine function, referred to here as skeletal muscle secretory dysfunction, defined as abnormal remodelling of the muscle secretome characterised by dysregulated myokine secretion and impaired muscle–bone–immune communication. As an endocrine organ, skeletal muscle releases bioactive factors involved in metabolic regulation, immune homoeostasis, and skeletal remodelling; therefore, altered secretion profiles may influence osteoimmune regulation [52]. Current evidence suggests that several bone‐related myokines are dysregulated in T2DM, although existing findings remain heterogeneous. Myostatin is generally elevated and associated with muscle atrophy, inflammation, and impaired bone remodelling [53]. Conversely, irisin, a potential osteoprotective myokine, is frequently reduced or exhibits impaired activity in T2DM, although substantial variability exists [54]. Whether these alterations directly drive skeletal deterioration or represent secondary adaptations remains unclear.

Collectively, skeletal muscle secretory dysfunction represents a pathological remodelling of the muscle endocrine network, characterised by impaired myokine‐mediated muscle–bone communication and increased inflammatory signalling. This concept provides a mechanistic framework for understanding how exercise may restore a healthier myokine profile and improve diabetic osteoimmune abnormalities.

### 2.2 T2DM‐Induced Cellular Dysfunction in the Bone Microenvironment

#### 2.2.1 T Cell Subset Imbalance

Evidence from experimental T2DM mouse models suggests that activated CD4^+^ T lymphocytes within the bone marrow may contribute to the local inflammatory microenvironment and may be associated with features of diabetic bone fragility [55]. Direct evidence from human bone marrow is still scarce, and current mechanistic understanding is therefore derived primarily from experimental models. Naive CD4^+^ T cells can differentiate into distinct subsets, including Th1, Th2, Th17, and regulatory T cells (Tregs) [56]. Among these, Th17 cells drive inflammatory responses through the secretion of interleukin‐17 (IL‐17), whereas Tregs suppress inflammation and maintain peripheral tolerance; the two subsets exhibit a reciprocal antagonism in both differentiation and function [57].

Under T2DM conditions, aberrant metabolic signals directly influence T cell differentiation. Th17 cells primarily rely on glycolysis for energy, whereas Tregs preferentially utilise fatty acid oxidation (FAO) and oxidative phosphorylation (OXPHOS), metabolic programs that are fundamental to their differentiation and functional maintenance [58, 59]. Consistent with this concept, Nicholas et al. reported that peripheral blood immune cells from individuals with T2DM exhibited a metabolic preference towards glycolysis and displayed enhanced Th17‐associated inflammatory signatures, suggesting that immunometabolic alterations and intercellular crosstalk may underlie systemic immune dysregulation in T2DM [60].

Moreover, evidence from experimental T2DM models and distinct clinical cohorts—including newly diagnosed T2DM and obesity‐associated T2DM—consistently indicates varying degrees of Th17/Treg imbalance. Xu et al. observed an elevated proportion of Th17 cells and a decreased proportion of Tregs in db/db diabetic mice [61]. Jiang et al. further demonstrated a reduced proportion of Tregs in the bone marrow of T2DM mouse models, accompanied by suppressed secretion of the anti‐inflammatory cytokines interleukin‐10 (IL‐10) and transforming growth factor‐β (TGF‐β) [62]. In clinical studies, newly diagnosed patients with T2DM likewise exhibited reduced circulating Treg frequencies and lower serum levels of IL‐10 and TGF‐β, with these alterations being more pronounced in individuals with obesity‐associated T2DM [63]. Meanwhile, enhanced Th17‐associated inflammatory responses have been observed in obesity‐associated T2DM and are closely associated with worsening glucose metabolic dysfunction [64]. Recent evidence further suggests that the gut–bone axis may represent an additional upstream regulator of Th17 activation in diabetic bone disease. Specifically, gut microbiota dysbiosis in hyperglycaemic mice promoted Th17 differentiation through a decanoic acid–IL‐17A axis, whereas microbiota restoration or IL‐17A blockade alleviated bone loss, supporting a potential role of the gut–bone axis in diabetic osteoimmune dysregulation [65]. However, whether this mechanism contributes to human T2DM‐associated bone disease remains to be determined.

IL‐17 can directly upregulate RANKL expression and downregulate OPG expression in osteoblasts/stromal cells, thereby promoting osteoclast differentiation and bone resorption [66]. In mice with preosteoclast‐specific deletion of IL‐17 receptor A (IL‐17RA), osteoclast numbers were reduced and trabecular microarchitecture was markedly improved [67]. In contrast, regulatory T (Treg) cells are recognized as important regulators of osteoimmune homoeostasis that suppress osteoclast formation and contribute to the maintenance of skeletal homoeostasis [68]. In addition, the anti‐inflammatory cytokines interleukin‐10 (IL‐10) and transforming growth factor‐β (TGF‐β) inhibit RANKL‐mediated osteoclast differentiation, thereby attenuating bone resorption [69, 70].

Although Th17/Treg imbalance is thought to promote osteoclastogenesis primarily through dysregulation of the RANKL/OPG axis, this mechanism should be interpreted within the broader pathological network of diabetic bone disease rather than as an isolated pathway. Chronic metabolic stress in T2DM not only promotes Th17/Treg immune imbalance but also concurrently activates AGE–RAGE signalling, induces osteocyte dysfunction, increases sclerostin expression, suppresses Wnt/β‐catenin signalling, and sustains local bone inflammation. Collectively, these alterations impair bone remodelling and compromise bone quality. Recent human bone tissue studies in postmenopausal women with T2DM or obesity further support a close association between local inflammatory activation, dysregulated Wnt signalling, and reduced bone strength [42], suggesting that osteoimmune dysregulation acts in concert with these pathogenic mechanisms to drive diabetes‐associated skeletal deterioration.

Taken together, the Th17/Treg balance represents an important component of osteoimmune regulation rather than a standalone pathogenic driver of diabetic bone disease [71]. In T2DM, Th17/Treg imbalance may be shaped by multiple upstream factors, including metabolic dysfunction, gut microbiota alterations, and local inflammatory cues, and may contribute to abnormal bone remodelling through disruption of the RANKL/OPG axis. However, this immune imbalance is more likely to interact with bone cell dysfunction and other pathological processes within the diabetic skeletal microenvironment, collectively influencing bone remodelling outcomes.

#### 2.2.2 Pro‐Inflammatory Polarization of Macrophages

In the context of T2DM, bone marrow macrophages (BMMs) act as crucial hubs linking metabolic dysregulation to imbalanced bone remodelling [72, 73]. Under physiological conditions, they maintain a dynamic equilibrium between pro‐inflammatory M1 and reparative M2 phenotypes, cooperatively regulating bone resorption and formation [74]. Under sustained hyperglycemia and metabolic disturbance, this balance shifts towards an M1‐dominant phenotype [75].

BMMs from db/db diabetic mice exhibit a pronounced pro‐inflammatory polarization, with an iNOS/CD206 fluorescence intensity ratio more than four‐fold higher than that of wild‐type controls, accompanied by elevated iNOS and IL‐1β expression and reduced CD206 expression [76]. In a high‐fat diet‐induced T2DM model, F4/80^+^ macrophages accumulate in the femoral bone marrow as early as week 4, preceding overt structural bone damage [77], suggesting that macrophage accumulation may be associated with early alterations in bone remodelling and inflammatory signalling during T2D‐related bone loss in experimental models.

The core mechanism perpetuating the M1 phenotype is immunometabolic reprogramming. At the metabolic level, M1 macrophages rely primarily on glycolysis, whereas M2 macrophages are more dependent on OXPHOS and FAO [78]. In the T2DM milieu, the expression of glucose transporter 1 (GLUT1) is upregulated, accelerating glucose uptake and thereby driving an elevated glycolytic rate, along with increased pentose phosphate pathway activity and concomitant accumulation of ROS [79, 80]. Impaired mitochondrial function leads to disruption at key nodes of the tricarboxylic acid (TCA) cycle, resulting in the accumulation of metabolic intermediates, particularly succinate. Succinate inhibits prolyl hydroxylase (PHD), thereby stabilising and activating the transcription factor hypoxia‐inducible factor‐1α (HIF‐1α) and promoting the expression of inflammatory cytokines such as IL‐1β, which metabolically locks macrophages in a pro‐inflammatory state [81]. More importantly, recent studies have proposed that a high‐glucose environment can induce a state of metabolic memory in bone marrow‐derived macrophages. Using a mouse model of diabetic fracture healing and in vitro cultures of murine BMMs, Zhang et al. demonstrated that even after normalisation of blood glucose, macrophages maintain high expression of CD86 and iNOS and persistently suppress osteogenesis and angiogenesis of bone marrow mesenchymal stem cells (BMSCs), owing to persistent histone modifications such as H3K4me3 [82]. These findings are primarily derived from animal models and in vitro experiments; whether similar epigenetic locking occurs in human T2DM BMMs, and whether it contributes to impaired fracture healing in patients, remains to be established. Nevertheless, this study provides an important mechanistic framework for understanding persistent impairment of bone repair under diabetic conditions.

Metabolically locked M1 macrophages contribute to bone remodelling imbalance through sustained secretion of TNF‐α, IL‐1β, and IL‐6, enhancing RANKL‐induced osteoclastogenesis while impairing osteoblast and endothelial function [83, 84, 85]. Under diabetic conditions, AGE‐induced NF‐κB activation further amplifies this pro‐inflammatory phenotype [45], reinforcing a chronic inflammatory microenvironment that disrupts bone homoeostasis.

Although direct evidence linking macrophage polarization to bone fragility in human T2D remains limited, human bone‐tissue studies report that inflammatory signatures are associated with suppressed Wnt/β‐catenin signalling and reduced bone strength [39, 41, 42]. Importantly, inflammatory signalling inhibits Wnt/β‐catenin activity while enhancing osteoclastogenic pathways, shifting bone remodelling towards bone loss. Together, these findings suggest that macrophage‐driven inflammatory remodelling links metabolic dysfunction to diabetic skeletal deterioration through coordinated regulation of Wnt/β‐catenin signalling and RANKL‐mediated osteoclastogenesis.

In summary, in T2DM, BMMs undergo M1 polarization characterised by glycolytic reprogramming and epigenetic locking. By secreting TNF‐α, IL‐1β, and IL‐6, they upregulate RANK receptor expression on osteoclast precursors and enhance RANKL‐induced osteoclastogenesis while suppressing the osteogenic ability of bone marrow mesenchymal stem cells (BMSCs), establishing a critical cellular basis for osteoimmune dysregulation.

#### 2.2.3 Imbalance of Osteogenic and Adipogenic Differentiation of BMSCs

BMSCs are a heterogeneous population of stromal progenitor cells with the capacity for osteogenic, adipogenic, and chondrogenic differentiation, and lineage commitment of these cells is fundamental for maintaining skeletal homoeostasis [86]. Experimental animal studies have demonstrated that BMSCs isolated from Zucker diabetic fatty (ZDF) rats exhibit markedly impaired clonogenicity, proliferation, and migration, accompanied by enhanced adipogenic differentiation and upregulation of peroxisome proliferator‐activated receptor γ (PPARγ) and AdipoQ [87]. Similarly, transcriptomic and functional analyses in high‐fat diet‐induced T2DM mouse models confirm that impaired BMSC proliferation and sustained suppression of osteogenic differentiation occur at early stages of diabetic bone loss [77]. Collectively, these findings support the idea that T2DM remodels the bone marrow microenvironment, thereby altering BMSC lineage commitment in a context‐dependent manner influenced by disease stage and local niche composition.

At the human level, Cassidy et al. reported that, although the osteogenic MSC subpopulation is significantly reduced in the bone marrow of T2DM patients, isolated bone marrow stromal cells retain normal proliferative, differentiative, and migratory capacities in vitro [88]. This suggests that dysfunction of bone marrow stromal cells in human T2DM more likely reflects sustained alterations in the diabetic bone marrow microenvironment rather than irreversible intrinsic defects, highlighting the importance of microenvironment‐dependent interpretation across species.

Mechanistically, PPARγ and Wnt/β‐catenin signalling act as key regulatory nodes governing BMSC lineage allocation in a dynamic antagonistic manner [89, 90]. Within the T2DM‐associated inflammatory milieu, pro‐inflammatory cytokines such as TNF‐α enhance PPARγ and C/EBPα expression, promoting adipogenic commitment of MSCs [91].

Increased adipogenic priming of BMSCs is closely linked to bone marrow adipose tissue (BMAT) expansion [92]. BMAT comprises constitutive (cBMAT) and regulated (rBMAT) components [93]. rBMAT is highly sensitive to metabolic status and undergoes pathological expansion under T2DM, altering the bone marrow niche and restricting osteogenic differentiation of BMSCs. Physiologically, BMAT participates in energy supply, hematopoietic support, and local bone marrow homoeostasis, with adaptive functions during skeletal growth or energy deprivation [94]. BMAT exhibits marked functional heterogeneity across skeletal sites, developmental stages, and metabolic conditions [95], underscoring the need to distinguish physiological from pathological BMAT expansion in disease interpretation.

Large‐scale population studies have provided genetic evidence for a potential causal relationship between BMAT and skeletal fragility. Using UK Biobank data (> 48,000 participants), Xu et al. showed that increased BMAT in the femoral shaft and total hip is potentially causally associated with osteoporosis, whereas T2DM–BMAT associations are site‐specific—positive in the spine but negative in femoral regions [96]. These findings highlight the spatial heterogeneity of BMAT and emphasise the importance of skeletal site specificity when interpreting its role in diabetic bone disease. Magnetic resonance spectroscopy further revealed that T2DM patients exhibit reduced marrow lipid unsaturation and increased saturation, compositional changes independently associated with fragility fractures irrespective of bone mineral density [97]. Thus, BMAT in T2DM should be viewed as part of a dynamic regulatory network rather than a uniform pathogenic determinant.

BMAT exerts lipotoxic and pro‐inflammatory effects on adjacent BMSCs and osteoblasts through secretion of free fatty acids (notably palmitic and stearic acids), cytokines, and adipokines [98, 99, 100]. Additionally, BMAT is a direct source of RANKL. Using Adipoq‐Cre–mediated conditional deletion, Hu et al. demonstrated that RANKL from bone marrow adipocyte lineage cells drives trabecular osteoclastogenesis and cancellous bone resorption; its selective deletion reduces osteoclast formation and increases trabecular bone mass [101]. Thus, BMSC lineage skewing promotes pathological BMAT expansion, and together they remodel the bone marrow niche towards RANKL/OPG imbalance and enhance osteoclastogenic activity through depletion of the MSC progenitor pool, secretion of pro‐inflammatory and pro‐osteoclastogenic mediators, and BMAT‐mediated RANKL signalling.

In summary, T2DM‐associated osteoimmune dysregulation may be conceptualised as a context‐dependent network linking metabolic stress, immune remodelling, and skeletal adaptation. Chronic metabolic perturbations disrupt bone marrow homoeostasis and create a pro‐inflammatory, lipotoxic milieu affecting multiple cellular compartments. Within this milieu, macrophage polarization, T cell imbalance, and BMSC lineage skewing appear to be coordinated by common inflammatory cues and may reciprocally interact through RANKL‐related pathways. Skeletal muscle‐derived signals may further modulate bone–immune crosstalk in a context‐dependent manner. These interconnected processes, driven by upstream metabolic stress, converge on RANKL/OPG dysregulation, reduced osteoblast activity, and enhanced bone resorption (Figure 1), providing a rationale for multimodal strategies such as exercise that target metabolic, immune, and musculoskeletal components.

**FIGURE 1:**
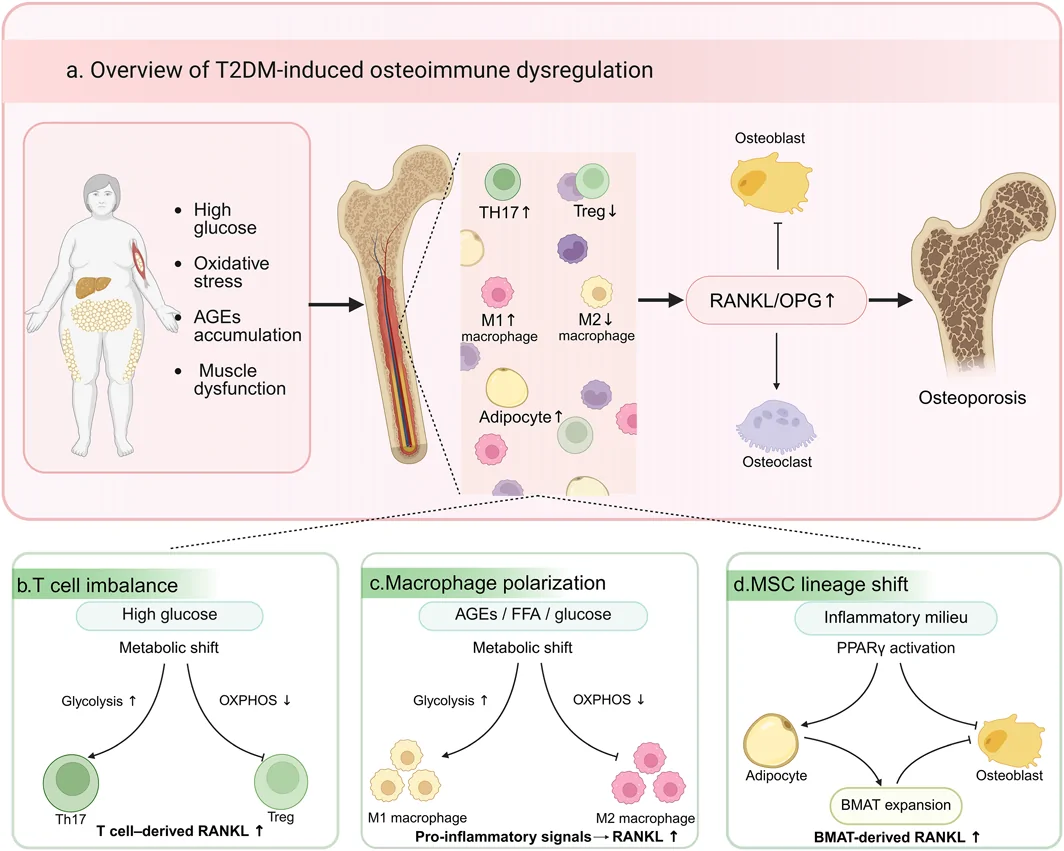
T2DM‐induced osteoimmune dysregulation in the bone marrow microenvironment. (a) T2DM‐associated metabolic drivers (hyperglycemia, AGEs, oxidative stress, muscle dysfunction) induce osteoimmune imbalance within the bone marrow microenvironment, characterised by a pro‐inflammatory shift (Th17, M1) and anti‐inflammatory suppression (Treg, M2), accompanied by adipogenic differentiation of mesenchymal stem cells and BMAT expansion. These changes elevate the RANKL/OPG ratio, promoting osteoclast activity and bone loss. (b–d) Detailed mechanisms underlying the cellular dysregulation depicted in (a). (b) Hyperglycemia promotes Th17 differentiation through glycolysis while suppressing Tregs via impaired oxidative phosphorylation. (c) AGEs and free fatty acids induce M1 polarization through glycolysis, while inhibiting M2 via impaired oxidative phosphorylation. (d) The inflammatory microenvironment activates PPARγ, skewing mesenchymal stem cells towards adipogenic differentiation and BMAT expansion. ↑, increase; ↓, decrease; AGEs, advanced glycation end products; BMAT, bone marrow adipose tissue; FFA, free fatty acids; MSC, mesenchymal stem cells; OPG, osteoprotegerin; PPARγ, peroxisome proliferator‐activated receptor γ; RANKL, receptor activator of nuclear factor‐κB ligand; Th17, t helper 17 cells; Treg, regulatory t cells.

## 3 Multidimensional Restoration of Diabetic Osteoimmune Homoeostasis by Exercise

### 3.1 Mechanical Stimulation Activates Osteogenic Signalling Pathways

Mechanical loading is a fundamental signal for maintaining bone mass. Although patients with T2DM may experience some degree of static loading due to increased body weight, this protective effect is often counteracted by elevated sclerostin levels, which suppress bone formation signalling [102]. Exercise‐generated mechanical loading and fluid shear stress can directly activate osteocytes, inhibit SOST gene transcription, and downregulate serum sclerostin levels, thereby relieving its inhibition of the Wnt/β‐catenin osteogenic pathway [103]. Human studies have demonstrated that canonical Wnt/β‐catenin signalling is reduced in T2DM bone and is associated with increased AGE accumulation and impaired bone strength, suggesting that impaired Wnt signalling represents an important feature of diabetic skeletal deterioration [41]. Although direct evidence from exercise intervention studies in humans with T2DM remains limited, findings from diabetic animal models provide mechanistic support for the involvement of Wnt/β‐catenin signalling in exercise‐mediated skeletal benefits. In T2DM mouse models, regular exercise has been shown to restore impaired Wnt/β‐catenin signalling and promote the differentiation of osteogenic precursor cells towards the osteoblastic lineage, thus improving trabecular microarchitecture and bone strength [104]. Mechanical activation of Wnt/β‐catenin signalling represents an important osteogenic response to exercise‐induced loading and contributes to the broader regulatory network underlying exercise‐mediated improvement of diabetic bone remodelling.

### 3.2 Exercise Ameliorates T2DM‐Associated Metabolic Abnormalities

In addition to direct mechanical stimulation, exercise indirectly modulates bone remodelling by improving the systemic metabolic environment. In T2DM, chronic hyperglycemia, AGE accumulation, and systemic low‐grade inflammation collectively constitute an important metabolic basis that compromises bone homoeostasis, and exercise can reverse this unfavourable milieu at multiple levels. Skeletal muscle contraction is one of the major physiological mechanisms for clearing circulating glucose [49]. Exercise promotes GLUT4 translocation and upregulates its expression, thereby enhancing glucose uptake capacity in skeletal muscle, ameliorating insulin resistance, and restoring glycaemic homoeostasis [105]. Consistent with these mechanisms, a recent randomized controlled trial demonstrated that combined aerobic and resistance exercise significantly improved glycaemic variability in patients with T2DM, further supporting the role of exercise in improving the systemic metabolic milieu that contributes to diabetic skeletal health [106]. Improved glycaemic control reduces hyperglycemia‐induced oxidative stress and AGE formation, thereby attenuating the sustained activation of inflammatory pathways by the AGE‐RAGE signalling axis [107].

Animal experiments have further demonstrated that long‐term aerobic exercise reduces pro‐inflammatory cytokine levels and enhances anti‐inflammatory responses in bone tissue in T2DM. For instance, in diabetic mouse models, regular exercise significantly decreases bone tissue levels of TNF‐α, lipocalin‐2 (LCN‐2), and oxidative stress‐related protein carbonylation, while increasing the anti‐inflammatory cytokine IL‐10, accompanied by improved bone microarchitecture [55]. Exercise also reduces visceral fat accumulation and modulates the adipokine secretory profile, lowering pro‐inflammatory factors such as TNF‐α, IL‐6, and leptin while elevating the anti‐inflammatory adipokine adiponectin, thus ameliorating adipose tissue inflammatory status [108, 109]. Numerous randomized controlled trials and meta‐analyses have also shown that regular exercise reduces circulating levels of C‐reactive protein (CRP), IL‐6, and TNF‐α in patients with T2DM, thereby alleviating the systemic chronic low‐grade inflammatory burden [110, 111].

### 3.3 Exercise Regulates the Osteoimmune Axis Through Myokines

As described in Section 2.1.3, diabetes can entrap skeletal muscle in a state of chronic inflammation and metabolic dysregulation, driving pathological alterations in the myokine secretory profile that weaken its protective regulatory influence on the immune system and bone tissue. Mounting evidence indicates that regular exercise can partially reverse this secretory dysfunction. Studies in T2DM patients and animal models have demonstrated that exercise elevates circulating levels of protective myokines, such as irisin and myonectin, while concurrently reducing the expression of negative regulators such as myostatin [112, 113]. Even under acute exercise conditions, individuals with T2DM retain a degree of secretory plasticity; a single exercise bout is sufficient to induce a transient rise in myokines and enhance glucose clearance [114]. These findings suggest that diabetic skeletal muscle has not entirely lost its secretory capacity but rather exists in a suppressed state that can be reactivated by exercise.

Myokines are a class of proteins and peptides actively secreted by skeletal muscle during contraction [115]. Once in the circulation, these molecules function as crucial signalling mediators between muscle and distant organs, forging a communication bridge among the skeleton, adipose tissue, and immune system [116, 117]. The major exercise‐responsive myokines involved in diabetic osteoimmune regulation are summarised in Table 1. Consequently, exercise not only restores muscle metabolic homoeostasis but also reactivates its endocrine function, reprogramming the immune microenvironment in a myokine‐dependent manner and ultimately influencing bone remodelling (Figure 2).

**TABLE 1:** Key mediators of exercise‐induced osteoimmune regulation.

Factor	Source	Exercise regulation	Primary target	Signalling pathway	Osteoimmune outcome	Primary axis	Validation in T2DM	Reference
IL‐6	Skeletal muscle	Acute↑; chronic resting↓	Macrophages; hepatocytes	IL‐6→IL‐10↑; IL‐1ra↑; TNF‐α↓	Context‐dependent anti‐inflammatory; potential RANKL regulation	Immune‐dominant	Partially confirmed	[118, 119, 120, 121, 122]
IL‐15	Skeletal muscle	Acute↑	T cells; NK cells; Tregs	PPARβ/δ↑; T‐cell oxidative metabolism↑	Treg support; potential limitation of Th17 polarization	Immune‐dominant	Inferred	[123, 124, 125, 126, 127, 128]
Irisin	Skeletal muscle	↑	Macrophages; BMSCs; BMAT adipocytes	TLR4/MyD88/NF‐κB↓; JAK2–STAT6–PPARγ↑; AMPK↑; Nrf2 ↑	M2 polarization↑; osteogenesis↑; Marrow adipogenesis↓	Metabolic–immune integrative	Partially confirmed	[129, 130, 131, 132, 133, 134]
BAIBA	Skeletal muscle	↑	WAT adipocytes; osteoclasts;	AMPK–PPARδ↑; SLC6A6–PI3K/AKT–NF‐κB↓; Nrf2↑	WAT browning↑; Osteoclast differentiation↓; lipotoxicity↓	Metabolic–immune integrative	Inferred	[135, 136, 137, 138, 139]
FGF21	Liver	Acute↑; chronic variable	Macrophages; adipocytes	NF‐κB↓; Nrf2↑	Anti‐inflammatory; antioxidant	Metabolic‐dominant	Partially confirmed	[140, 141, 142, 143, 144, 145, 146, 147]
Myonectin	Skeletal muscle	↑	Hepatocytes; adipocytes	FA uptake↑; NEFA↓	Lipotoxic inflammation↓(indirect)	Metabolic‐dominant	Inferred	[148, 149]
Myostatin	Skeletal muscle	↓	Osteoblasts; osteoclasts; macrophages	ActRIIB–Smad2/3↑; NF‐κB/MAPK↑; CCL20↑	Osteoblast differentiation↓; osteoclastogenesis↑; Pro‐inflammatory polarization	Metabolic–Immune–Mechanical	Partially confirmed	[150, 151, 152]

**FIGURE 2:**
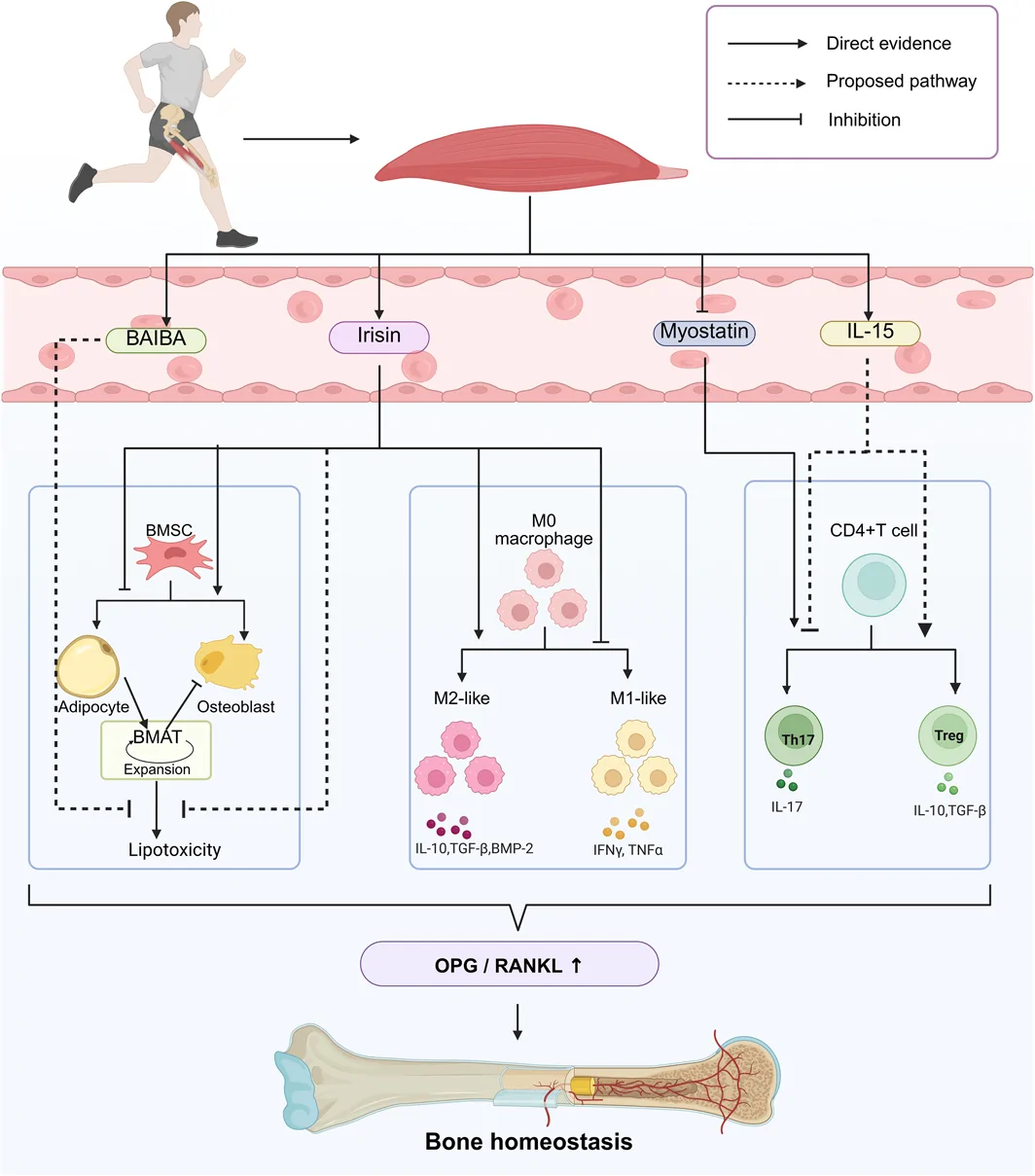
Exercise–myokine–osteoimmune axis in diabetic bone disease. Exercise induces the secretion of myokines (irisin, BAIBA, IL‐6, IL‐15) while suppressing myostatin. These myokines promote M2 macrophage polarization, Treg differentiation, and osteogenic differentiation of mesenchymal stem cells, while inhibiting M1 polarization, Th17 differentiation, and adipogenic differentiation of mesenchymal stem cells. Irisin and BAIBA also improve the metabolic function of bone marrow adipose tissue, thereby alleviating lipotoxicity. Collectively, these effects restore the RANKL/OPG balance and bone homoeostasis. ↑, increase; ↓, decrease; BAIBA, β‐aminoisobutyric acid; BMP‐2, bone morphogenetic protein‐2; BMSC, bone marrow mesenchymal stem cells; IFN‐γ, interferon‐γ; IL‐5, interleukin‐5; IL‐10, interleukin‐10; IL‐17, interleukin‐17; OPG, osteoprotegerin; RANKL, receptor activator of nuclear factor‐κB ligand; TGF‐β, transforming growth factor‐β; TNF‐α, tumour necrosis factor α.

#### 3.3.1 The Anti‐Inflammatory Cascade Initiated by Muscle‐Derived IL‐6

In the context of the osteoimmune axis, muscle‐derived IL‐6 has been proposed to initiate a rapid anti‐inflammatory cascade that may influence the RANKL/OPG signalling milieu. Unlike the sustained secretion of IL‐6 from immune cells during chronic inflammation, exercise‐induced IL‐6 exhibits a transient pulsatile elevation. For instance, following a marathon race, plasma IL‐6 concentrations can rise approximately 100‐fold above resting levels [153]. During prolonged exercise, the net release of IL‐6 from skeletal muscle may reach 17 times the magnitude of the arterial IL‐6 increase, indicating that contracting muscle is the main source of circulating IL‐6 [154, 155]. Importantly, this acute IL‐6 exerts a well‐defined anti‐inflammatory effect [118].

Acute exercise‐induced IL‐6 elevation initiates an anti‐inflammatory cascade by upregulating IL‐10 and interleukin‐1 receptor antagonist (IL‐1Ra) [119], while concurrently suppressing TNF‐α production [120]. IL‐1Ra exerts its anti‐inflammatory action by competitively binding to the IL‐1 receptor and blocking the pro‐inflammatory signals of IL‐1α and IL‐1β [121]. Multiple clinical studies have demonstrated that recombinant IL‐1Ra improves glycaemic control and β‐cell secretory function and reduces systemic inflammatory markers in both diabetic animal models and patients [156, 157, 158]. Previous work has established IL‐10 as a potent anti‐inflammatory cytokine [159]. The hexameric complex formed by IL‐10 with IL‐10Rα/β initiates signal transduction [160] and attenuates obesity‐induced inflammation by suppressing macrophage TNF‐α production [161], promoting polarization towards the M2 phenotype [162], and alleviating adipose tissue inflammation and hepatic steatosis through targeted delivery to macrophages [163]. Recent research has revealed that IL‐10 epigenetically targets interferon regulatory factor (IRF) transcription factors to suppress the expression of NF‐κB target genes and interferon‐stimulated genes [164].

At the bone metabolism level, accumulating evidence from cellular and animal studies indicates that IL‐1Ra and IL‐10 can regulate osteoclast differentiation and bone remodelling. In vitro studies have confirmed that IL‐1β downregulates OPG expression in periodontal ligament fibroblasts, whereas IL‐1RA upregulates OPG expression and inhibits IL‐1β‐mediated osteoclast differentiation [165]. Mice deficient in IL‐1RA exhibit a two‐fold increase in the number of osteoclast precursors in long bones and jaws, with markedly enhanced osteoclastogenesis and bone resorption capacity [166]. IL‐10 has been shown to directly modulate osteoclast differentiation in experimental models. It has been shown to significantly suppress RANKL‐induced osteoclast differentiation and downregulate the key osteoclast transcription factor nuclear factor of activated T cells c1 (NFATc1) [69], and in T2DM‐mimicking bone defect animal models, it attenuates inflammation and promotes new bone formation [167]. These findings, largely derived from systemic anti‐inflammatory responses, in vitro osteoclast studies, and non‐diabetic models, suggest that the anti‐inflammatory cascade initiated by muscle‐derived IL‐6 may contribute to the regulation of bone remodelling through downstream mediators such as IL‐1Ra and IL‐10, although direct evidence demonstrating this pathway within the diabetic bone microenvironment remains limited. In contrast to the acute elevation, long‐term regular training reduces resting IL‐6 levels in T2DM patients [122]. This dual regulation of IL‐6 by exercise—initiating an anti‐inflammatory cascade during acute exercise while reducing basal inflammatory tone with long‐term training—may contribute to a more favourable osteoimmune environment over time, although its specific contribution to T2DM‐associated bone remodelling requires further investigation.

#### 3.3.2 Irisin Promotes M2 Polarization and Improves BMAT Function

In the pathological context of T2DM, BMMs undergo a pro‐inflammatory phenotypic shift, continuously secreting inflammatory cytokines such as TNF‐α, IL‐1β, and IL‐6 that promote osteoclastogenesis and suppress osteogenesis [76]. Exercise has been demonstrated to restore M1/M2 macrophage balance and alleviate metabolic inflammation [168]. Chronic moderate‐intensity exercise attenuates lipopolysaccharide (LPS)‐stimulated NF‐κB activation and pro‐inflammatory gene expression in BMMs, while simultaneously enhancing their reliance on oxidative phosphorylation and mitochondrial mass, accompanied by altered chromatin accessibility at inflammation‐related genes, suggesting that exercise cultivates an anti‐inflammatory phenotype through metabolic and epigenetic reprogramming [169].

Irisin is a myokine secreted by skeletal muscle during exercise and proteolytically cleaved from fibronectin type III domain‐containing protein 5 (FNDC5) under the transcriptional control of peroxisome proliferator‐activated receptor‐γ coactivator‐1α (PGC‐1α) [170]. In newly diagnosed T2DM patients, serum levels of irisin are decreased and positively correlate with the bone formation markers procollagen type 1 N‐terminal propeptide (P1NP) and osteocalcin [54].

In RAW264.7 macrophages, irisin suppresses the TLR4/MyD88/NF‐κB pathway, thereby reducing the expression of pro‐inflammatory factors such as IL‐1β, TNF‐α, IL‐6, and monocyte chemoattractant protein‐1 (MCP‐1), and induces a phenotypic switch from M1 to M2 [130, 171]. Ye et al. reported that irisin treatment polarises both M0 and M1 macrophages towards an M2 phenotype, characterised by upregulation of CD206, arginase‐1 (ARG‐1), and transforming growth factor‐β1 (TGF‐β1) expression, accompanied by activation of AMP‐activated protein kinase (AMPK) signalling [131]. Tu et al. further revealed that irisin binds to integrin αVβ5, activating JAK2‐STAT6 signalling, which promotes the transcription of anti‐inflammatory genes mediated by PPARγ and Nrf2, thereby driving M2 polarization [132]. Rahman et al. confirmed in an inflammatory mouse model that irisin treatment markedly upregulates M2 markers IL‐4 and IL‐10 while downregulating M1 markers IL‐1β, IL‐6, and TNF‐α [133].

Macrophages reprogrammed by irisin can further promote bone regeneration. For instance, co‐culture experiments with RAW264.7 macrophages and MC3T3‐E1 cells demonstrated that conditioned medium from irisin‐treated M2 macrophages significantly enhances osteoblast differentiation, as evidenced by increased ALP activity and enhanced mineralised nodule formation [131]. M2 macrophages can suppress osteoclast formation and promote bone formation by secreting osteogenesis‐related proteins such as bone morphogenetic protein‐2 (BMP‐2), TGF‐β, and IL‐10 [167, 172, 173].

The bone‐protective effects of irisin have been further validated in models of metabolic dysregulation. In a high‐fat diet‐induced model, FNDC5 deficiency exacerbates bone marrow inflammation and suppresses osteogenesis of MSCs, whereas irisin supplementation inhibits TLR4/NF‐κB signalling and improves the bone marrow microenvironment [134]. Moreover, in T2DM mice, irisin attenuates bone loss by modulating inflammasome‐related pathways [129].

In addition, irisin has been implicated in the regulation of bone marrow adiposity. FNDC5 deletion aggravates bone marrow fat accumulation in high‐fat diet‐fed mice, whereas exogenous irisin suppresses adipogenic differentiation of BMSCs and promotes osteogenic differentiation [134]. Coupled with extensive evidence that irisin induces the expression of browning genes such as uncoupling protein 1 (UCP‐1) and PGC‐1α in white adipose tissue [170, 174], irisin may endow BMAT with a higher oxidative metabolic capacity.

Collectively, exercise‐induced irisin can ameliorate the bone marrow immune microenvironment and restore the balance between bone resorption and formation by promoting macrophage M2 polarization, suppressing inflammatory signalling, and enhancing BMAT metabolic capacity (Figure 2), representing an important osteoimmune mechanism underlying exercise‐mediated improvement of diabetic bone disease.

#### 3.3.3 IL‐15 and IL‐7 Modulate the Th17/Treg Balance

IL‐15 can be secreted by skeletal muscle in response to exercise [123]. A meta‐analysis has shown that acute exercise significantly elevates circulating IL‐15 levels, which remain elevated for at least one hour [124], and animal studies have likewise confirmed that exercise upregulates IL‐15 expression in skeletal muscle [125].

At the metabolic level, IL‐15 has been demonstrated to promote a preferential reliance on oxidative phosphorylation in memory T cells through activation of the PPARβ/δ pathway [175]. Given that Th17 differentiation is highly dependent on glycolysis, this metabolic bias may theoretically limit glycolysis‐driven Th17 polarization, thereby indirectly restoring the Th17/Treg balance. Accordingly, IL‐15 may represent a potential exercise‐responsive metabolic immunomodulator. By promoting oxidative metabolism in T cells, IL‐15 has been proposed to influence T‐cell fate decisions, although whether this mechanism contributes directly to restoration of the Th17/Treg balance in diabetic bone disease remains to be established.

At the immune microenvironment level, IL‐15 not only maintains the homoeostasis of NK cells and CD8^+^ memory T cells [126] but also supports the survival of specific regulatory T cell subsets in the bone marrow. For example, Pulvirenti et al. demonstrated that the survival of bone marrow EOMES^+^ Tr1‐like regulatory T cells is highly dependent on IL‐15 signalling [127]. These cells secrete IL‐10 and maintain local immune tolerance; therefore, IL‐15 may indirectly contribute to a more favourable bone remodelling by supporting regulatory T‐cell function.

In contrast to the functional remodelling exerted by IL‐15, IL‐7 primarily serves a systemic role in maintaining T cell homoeostasis. Studies have confirmed that skeletal muscle can express and secrete IL‐7 [176] and that its serum levels decline with age but can be partially preserved by exercise [177]. IL‐7 Rα signalling has been shown to exert tissue‐specific effects on Treg maintenance in metabolic tissues [178]. Moreover, Tregs expanded with a combination of IL‐7 and IL‐15 exhibit a more naïve phenotype, enhanced metabolic fitness, prolonged in vivo survival, and more potent immunosuppressive function [128]. Therefore, IL‐7 may primarily act as a supportive factor, potentially providing the cellular substrate for IL‐15‐mediated functional remodelling.

In summary, IL‐15 and IL‐7 may represent complementary exercise‐responsive cytokines with the potential to influence adaptive immune remodelling. IL‐15 has been proposed to support regulatory T‐cell function through metabolic and bone marrow microenvironmental mechanisms, whereas IL‐7 may primarily contribute to T‐cell maintenance. Their coordinated actions could potentially favour restoration of the Th17/Treg balance and indirectly influence the RANKL/OPG axis (Figure 2). However, current evidence is derived predominantly from non‐T2DM models, and whether these cytokines directly regulate osteoimmune homoeostasis in diabetic bone disease remains to be established.

#### 3.3.4 BAIBA Targets Metabolic Remodelling of Bone Marrow Adipose Tissue

BMAT is not merely a space‐filling tissue but a metabolically active, insulin‐sensitive, unique fat depot that intrinsically possesses an inducible brown adipose‐like phenotype, as evidenced by high‐level expression of brown fat characteristic genes such as *Prdm16*, *PGC‐1α*, and *Dio2* [136]. However, its oxidative metabolic capacity and thermogenic gene expression decline substantially during ageing and in T2DM [136]. Clinical studies have further demonstrated that insulin‐stimulated glucose uptake in femoral BMAT is impaired in obese and T2DM patients, yet partially restored after bariatric surgery, underscoring the functional plasticity of BMAT [179].

β‐aminoisobutyric acid (BAIBA) is a small‐molecule myokine secreted in response to exercise or skeletal muscle PGC‐1α overexpression [137]. Seminal work has established that BAIBA induces the expression of brown/beige adipocyte‐specific genes in white adipose tissue and enhances fatty acid β‐oxidation capacity [135]. Subsequent studies revealed that BAIBA activates the AMPK–PPARδ pathway to drive the transcription of downstream fatty acid oxidation‐related genes such as *Cpt1*, *Acox1*, and *Fabp3*, thereby reducing the accumulation of lipotoxic intermediates [138]. The most recent evidence indicates that exercise‐induced BAIBA, through the SLC6A6 receptor, inhibits the PI3K/AKT/NF‐κB inflammatory pathway, activates the NRF2‐mediated antioxidant system, and downregulates the expression of key osteoclast genes, thus suppressing osteoclast differentiation [139]. Although direct evidence for BAIBA action in BMAT remains limited, its established roles in adipose browning and bone protection suggest that it may improve the bone marrow microenvironment by enhancing oxidative metabolism and reducing inflammatory output.

In summary, exercise‐induced BAIBA may promote BMAT oxidative metabolism and suppress inflammatory signalling, thereby potentially contributing to a more favourable bone marrow microenvironment and bone remodelling. However, direct evidence supporting these effects within T2DM BMAT and the diabetic bone marrow microenvironment remains limited.

#### 3.3.5 Myostatin Downregulation Relieves Negative Regulation of Osteogenesis

Myostatin (GDF‐8), a member of the TGF‐β superfamily, was initially identified as a negative regulator of muscle growth [180]. In patients with T2DM, skeletal muscle myostatin mRNA expression has been reported to be approximately 1.4‐fold higher than that in healthy controls [181]. Alterations in circulating myostatin levels have also been observed in some clinical populations, such as elderly men with T2DM, although the magnitude and consistency of these changes may vary depending on metabolic status and patient characteristics [182].

At the inflammatory level, myostatin has been proposed as a potential amplifier of inflammatory signalling, although direct evidence in diabetic bone or bone marrow remains limited. Studies from non‐diabetic inflammatory and catabolic models have shown that TNF‐α can induce myostatin expression through NF‐κB‐dependent pathways [183], whereas myostatin may further enhance inflammatory cytokine production, including TNF‐α and IL‐1β, through PI3K/Akt/AP‐1 signalling in inflammatory cell or tissue models [150, 184]. Myostatin has been reported to promote oxidative stress responses through ROS generation and NADPH oxidase 4 (NOX4) activation in osteogenic and metabolic disease models, thereby impairing osteogenic activity and contributing to an increased RANKL/OPG ratio in bone‐related cellular models [185, 186]. Furthermore, inflammatory arthritis studies suggest that myostatin may regulate CCL20–CCR6‐mediated Th17 cell recruitment, indicating a potential role in immune amplification [187]. Collectively, these findings support the concept that myostatin may function as a component of a broader inflammatory amplification network; however, whether this network directly contributes to T2DM‐associated bone immune dysregulation remains to be determined.

Beyond its immunomodulatory effects, myostatin can also directly act on bone lineage cells. By binding to the ActRIIB receptor, it activates the Smad2/3 pathway and suppresses the expression of Runx2 and Osterix, thereby inhibiting osteogenic differentiation [188, 189, 190]. Meanwhile, myostatin promotes NFATc1 nuclear translocation via Smad2 and enhances osteoclast differentiation through the NF‐κB/MAPK pathway [151, 191]. In diabetic animal models, myostatin antibody treatment improves bone defect repair [152]. Therefore, these findings suggest that myostatin may contribute to bone remodelling imbalance in T2DM‐related bone disease through combined effects on inflammatory amplification and direct regulation of bone lineage cells, although the relative contribution of each pathway remains to be clarified.

Exercise intervention has been demonstrated to significantly reduce myostatin levels in both muscle and the circulation of T2DM patients [182, 192]. At the molecular level, the exercise‐induced myokine decorin (DCN) directly binds myostatin and prevents its interaction with the ActRIIB receptor, thereby inhibiting downstream Smad2/3 signalling; concomitantly, exercise increases circulating follistatin levels, which may further contribute to suppression of myostatin activity [193].

In summary, myostatin may represent a pathological mediator linking metabolic stress, inflammation, and impaired bone remodelling. Exercise‐induced downregulation of myostatin expression and activity may contribute to a more favourable osteoimmune and bone‐remodelling environment through its effects on inflammatory signalling and bone metabolic pathways. However, whether exercise‐mediated myostatin suppression directly restores RANKL/OPG signalling in T2DM bone remains to be determined.

### 3.4 Exercise‐Related Metabolic Regulators and Their Potential Links to Osteoimmune Regulation

Beyond classical muscle‐derived signals, exercise may influence systemic metabolic and inflammatory states through metabolism‐related regulators, which may indirectly influence osteoimmune regulation by modifying systemic metabolic and inflammatory states. Fibroblast growth factor 21 (FGF21) is a metabolic hormone predominantly derived from the liver and adipose tissue, and is more appropriately considered a hepatokine rather than a classical myokine [194]. Exercise has been reported to increase circulating FGF21 levels, including in individuals with T2DM, suggesting that exercise‐induced FGF21 responses may participate in metabolic adaptation [141, 192].

Circulating FGF21 levels are frequently elevated in T2DM, which is generally considered a compensatory response to metabolic stress [142, 195]. However, chronic inflammation may impair FGF21 signalling through suppression of its co‐receptor β‐Klotho, as TNF‐α has been shown to reduce β‐Klotho expression via the JNK1 pathway [140]. FGF21 possesses anti‐inflammatory and antioxidant properties through modulation of NF‐κB and Nrf2‐related pathways in metabolic and inflammatory disease models [144, 145], and these effects could, in principle, contribute to a less inflammatory systemic environment that may be relevant to osteoimmune regulation. However, direct evidence for FGF21‐mediated bone protection in the diabetic bone microenvironment is currently lacking. In fact, the bone effects of FGF21 are context‐dependent: persistent pathological elevation has been associated with impaired bone metabolism in some models, whereas exercise‐induced increases may exert beneficial effects predominantly through systemic metabolic improvement, although direct bone effects remain uncertain [146, 147].

Myonectin (CTRP15) is a skeletal muscle‐derived regulator of lipid homoeostasis that promotes fatty acid uptake in metabolic tissues, potentially limiting circulating lipid overload and lipotoxic inflammatory responses [196]. Mechanistically, myonectin has been implicated in lipid metabolic regulation, although the precise mechanisms underlying its exercise responsiveness remain under investigation [148]. Exercise has been reported to increase myonectin levels, accompanied by improvements in insulin sensitivity and lipid metabolism [149]. However, alterations in circulating myonectin levels in T2DM remain controversial, with studies reporting either elevated or reduced levels in different patient populations [197, 198]. These inconsistent findings are likely attributable to differences in disease stage, glycaemic control, obesity status, and methodological heterogeneity across studies. Overall, current evidence primarily supports a metabolic regulatory role for myonectin, whereas its direct involvement in diabetic bone remodelling and osteoimmune regulation remains unclear.

In summary, FGF21 and myonectin should be considered as exercise‐responsive metabolic regulators rather than established osteoimmune mediators. Their potential relevance to diabetic bone disease, which may primarily arise from their effects on systemic metabolic homoeostasis and inflammatory status, remains to be determined.

### 3.5 Disease‐Specific Responses and Evidence Gaps in Exercise‐Mediated Osteoimmune Regulation in T2DM

Although exercise has been widely demonstrated to improve bone health and reduce fracture risk, its skeletal protective mechanisms in individuals with T2DM may not simply represent an extension of those observed in non‐diabetic populations. In healthy individuals, exercise‐induced skeletal benefits are largely mediated by mechanical loading and physiological bone remodelling. However, T2DM is characterised by chronic low‐grade inflammation (metaflammation), impaired osteoimmune homoeostasis, BMAT expansion, and altered myokine secretion profiles. Moreover, hyperglycemia and AGEs accumulation may impair osteogenic signalling and osteocyte mechanosensitivity, potentially reducing the anabolic response to mechanical stimuli. Therefore, in T2DM, exercise may provide skeletal benefits not only through mechanical stimulation but also through regulation of muscle‐derived factors that modulate inflammatory and immune environments (Figure 3). Exercise‐responsive myokines, including irisin, IL‐6, IL‐15, and myostatin, may therefore represent important complementary mediators of skeletal adaptation under diabetic conditions. The altered exerkine response observed in T2DM compared with healthy individuals further suggests that exercise partially corrects pathological signalling disturbances rather than merely induces physiological adaptations [199].

**FIGURE 3:**
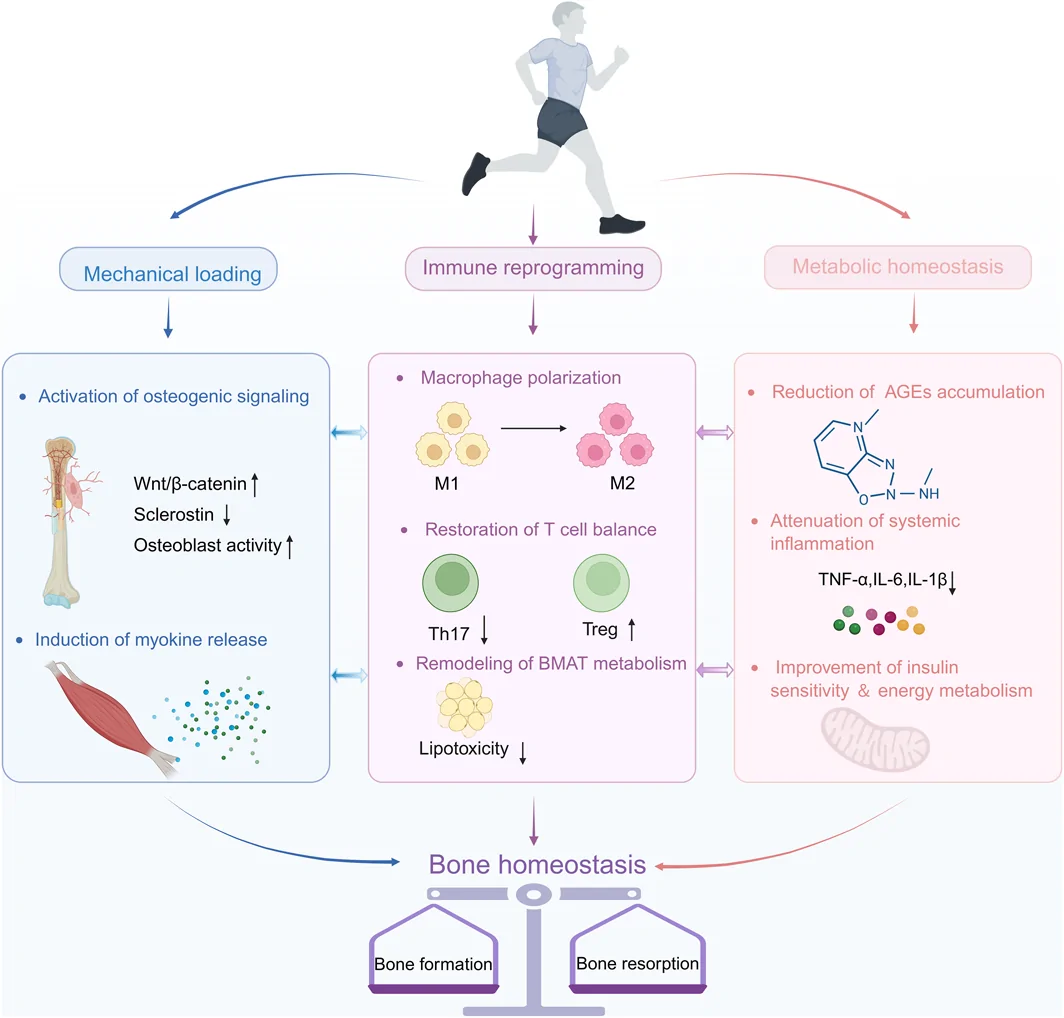
Exercise regulates bone homoeostasis through mechanical–metabolic–immune multi‐axis coupling. The mechanical, metabolic, and immune regulatory axes are reciprocally coupled, orchestrating osteoblast and osteoclast activities through multi‐level signal integration and coordinating the RANKL/OPG balance to maintain bone homoeostasis. ↑, increase; ↓, decrease; AGEs, advanced glycation end products; IL‐1β, interleukin 1β; IL‐6, interleukin‐6; Th17, T helper 17 cells; TNF‐α, tumour necrosis factor α; Treg, regulatory T cells.

Exercise modality may also influence myokine responses and skeletal outcomes in T2DM. Aerobic, resistance, and combined exercise interventions have all demonstrated metabolic benefits; however, their effects on exercise‐responsive factors, such as irisin, myostatin, and FGF21, as well as skeletal parameters, are not fully comparable (Table 2). Current evidence suggests that combined training may provide potential advantages by integrating metabolic regulation and mechanical loading. Nevertheless, direct head‐to‐head clinical trials comparing exercise modalities based on bone outcomes remain limited, and the optimal exercise prescription for T2DM‐related bone disease remains to be established.

**TABLE 2:** Differential effects of exercise modalities on myokine profiles and skeletal outcomes in T2DM.

Exercise modality	Population/mode	Myokine or inflammatory responses	Skeletal outcomes	Key references
Aerobic training	Women with T2DM (within different BMI categories)	Irisin↑; myonectin unchanged	Not assessed	[3]
	T2DM patients	IL‐1β, IL‐8, IL‐10 and IL‐15 altered	Not assessed	[200]
	T2DM mice	No myokine analysis; TNF‐α↓, IL‐10↑, LCN‐2↓	BV/TV↑; Tb.Sp↓; cortical density↑; fracture resistance↑	[55]
HIIT	STZ diabetic rats	FNDC5↑, myonectin↑	Not assessed	[112]
Resistance training	Elderly men with/without T2DM	Myostatin↓, FGF‐21↓	Not assessed	[182]
	T2DM patients	Not assessed	P1NP↑34%, osteocalcin↑31%, CTX↑36%; BMD maintained	[17]
Combined training	Men with T2DM	Irisin↑, follistatin↑, myostatin↓	Not assessed	[192]
	Older adults with T2DM	Not assessed	BMC↑; functional capacity improved	[18]
	Older adults with T2DM	Not assessed	Lumbar spine, femoral neck and total hip BMD↑; TBS↑	[201]

Importantly, current evidence supporting the exercise–myokine–osteoimmune axis is derived from studies across different experimental levels. Some myokines have received support from T2DM‐related human or experimental studies, whereas others are mainly inferred from non‐diabetic models or in vitro investigations (Table 1). For example, exercise‐induced changes in irisin and IL‐6 have been demonstrated in human studies, while their direct effects on osteoimmune remodelling are primarily supported by mechanistic studies. In contrast, the roles of IL‐15 and BAIBA in diabetic bone disease remain insufficiently characterised. Furthermore, no study has simultaneously examined exercise‐induced myokine alterations, osteoimmune remodelling, and skeletal outcomes within the same T2DM model. Whether exercise induces quantitatively or mechanistically distinct skeletal benefits in T2DM compared with non‐diabetic populations therefore remains an important unresolved question.

## 4 Summary and Outlook

Under T2DM conditions, alterations in the bone marrow immune microenvironment, metabolic homoeostasis, and skeletal muscle‐derived signalling profiles have been proposed to contribute to impaired bone remodelling and increased skeletal fragility. Exercise may counteract these pathological changes through the coordinated regulation of mechanical loading, metabolic adaptation, and myokine‐mediated signalling. Mechanical stimuli directly promote osteogenic responses, whereas skeletal muscle contraction induces the release of exercise‐responsive myokines. Improvements in systemic metabolic homoeostasis may reduce AGE accumulation, oxidative stress, and inflammatory burden, thereby creating a more favourable environment for bone marrow function. Exercise‐induced myokines, including IL‐6, irisin, BAIBA, and IL‐15, together with suppression of myostatin signalling, may participate in regulating macrophage polarization, T cell responses, BMAT metabolism, and osteogenic–osteoclastic coupling, potentially contributing to osteoimmune balance and RANKL/OPG regulation. Metabolic regulators such as FGF21 and myonectin may further influence the osteoimmune microenvironment through modulation of lipid metabolism and inflammatory responses. Collectively, the emerging exercise‐responsive myokine–osteoimmune axis provides a conceptual framework for understanding how skeletal muscle activity may interact with metabolic and immune regulation to influence skeletal health in T2DM.

Future studies should focus on establishing the causal relationship between exercise‐induced molecular adaptations and skeletal outcomes in T2DM. Longitudinal studies integrating exercise interventions with dynamic profiling of myokines and immune alterations are needed to identify key mediators of skeletal adaptation and determine whether diabetic conditions modify exercise responsiveness. Mechanistic investigations combining genetic manipulation or pharmacological modulation of specific myokines with comprehensive assessment of immune phenotypes, RANKL/OPG signalling, bone microarchitecture, and biomechanical properties will be essential to validate the functional significance of the proposed pathways. Furthermore, future clinical studies should incorporate multi‐dimensional skeletal assessment, including imaging‐based parameters, bone turnover markers, high‐resolution peripheral quantitative CT (HR‐pQCT), trabecular bone score (TBS), bone material strength index (BMSi), microindentation‐based evaluation, and fracture outcomes, to better characterise changes in bone quality beyond conventional BMD measurements. Importantly, clinical translation should consider individual variability, including age, sex, disease duration, metabolic control, diabetic complications, and medication use, to develop personalised exercise strategies for preventing and managing T2DM‐related bone disease.

## Author Contributions

Chao Wu drafted the manuscript and created the figures using Biorender.com. Jun Zou and Yu Yuan critically revised the manuscript and figures.

## Funding

This work is supported by the National Natural Science Foundation of China (No. 81901430), the Guangdong Philosophy and Social Science Foundation Regular Project (No. GD23YTY04), the Special Projects in Key Fields of Universities in Guangdong Province (No. 2024ZDZX2059, 2025ZDZX2039) and the Shanghai Key Lab of Human Performance (Shanghai University of Sport) (No. 11DZ2261100).

## Conflicts of Interest

The authors declare no conflicts of interest.

## Data Availability

The authors have nothing to report.
